# Gluteal Augmentation: A Historical Perspective on Aesthetic Practice

**DOI:** 10.1093/asjof/ojae124

**Published:** 2024-12-12

**Authors:** Roberto Chacur

## Abstract

Buttock augmentation has emerged as a significant focus in cosmetic surgery, driven by advancements in techniques and increasing patient interest in body contouring. The evolution of this field, from early pioneering methods to modern, diverse approaches, highlights the need to understand the specific characteristics of each technique and their implications for aesthetic outcomes. The author aims to provide a detailed review of 4 major buttock augmentation techniques: gluteal implants, Brazilian butt lift (BBL), intramuscular polymethylmethacrylate (PMMA), and deep subcutaneous hyaluronic acid fillers. The goal is to assess the benefits and limitations of each method, helping practitioners and patients make informed decisions tailored to their preferences and needs. A comprehensive literature review was conducted, incorporating clinical studies, case reports, and expert opinions on these 4 techniques. Evaluation criteria included effectiveness, safety, recovery time, and patient satisfaction. Data were synthesized to provide a comparative analysis of each method. Gluteal implants offer predictable volume but involve surgical risks and lengthy recovery. The BBL, using autologous fat, delivers natural results and body contouring benefits but carries risks such as fat embolism and fat reabsorption. Intramuscular PMMA fillers provide permanent results with minimally invasive application but are challenging to remove. Hyaluronic acid fillers are reversible and minimally invasive but require periodic maintenance and may present risks like material migration. In this review, the author highlights the advantages and drawbacks of each technique, emphasizing individualized assessments and practitioner expertise. By outlining these methods, the author supports informed decision making in buttock augmentation.

**Level of Evidence: 5 (Therapeutic):**

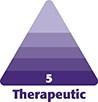

Buttock augmentation is one of the most popular aesthetic practices today, with deep historical roots in art and culture. Since ancient times, depictions of the gluteal anatomy in paintings and sculptures have reflected society's admiration for the female form. For example, the “Venus Callipyge,” an ancient Greek statue dating back to the second century BCE, explicitly celebrates the beauty of the female buttocks.

These artistic representations symbolize not only physical beauty but also fertility, sexuality, and evolving cultural ideals, influencing modern aesthetic practices. Today's techniques for buttock augmentation, including biostimulants and surgical options, continue this legacy, enhancing appearance and boosting self-confidence. The pursuit of buttock augmentation reflects cultural beauty ideals and personal self-expression.

The evolution of gluteal augmentation has been marked by significant technological advancements. The first subcutaneous gluteal implant surgery was performed by Dr Bartels in 1969, using silicone implants initially designed for breast augmentation.^1^ This pioneering procedure advanced surgical techniques for enhancing buttock shape and volume and established a foundation for future innovations, documenting outcomes and early complications.

From 1973 to 1975, notable case reports emerged from Cocke, Ricketson, Douglas, and Bartels, laying the groundwork for liposuction, pioneered by Giorgio Fischer between 1975 and 1976, which later influenced fat grafting techniques.^2-5^ In the 1980s, Dr Yves-Gérard Illouz published studies on fat grafting, leading to autologous fat remodeling, or lipoaugmentation.^6,7^

In 1984, José Robles introduced the submuscular implant technique, and José Abel de la Peña developed the subfascial approach.^8,9^ These innovations improved aesthetic outcomes. Rafael Vergara introduced intramuscular implants in 1996,^10^ followed by Gonzales's XYZ technique for implant placement in 2004.^11^ More recently, from 2019 to 2024, a multicentric study by Roberto Chacur provided valuable insights into intramuscular gluteal filling.^12-14^

Throughout the years, gluteal augmentation has evolved to incorporate more sophisticated techniques, such as autologous fat transfer and fillers like polymethylmethacrylate (PMMA), which have become widely used in clinical practice.^13-15^ Although early methods, such as subcutaneous implants, were relatively straightforward, modern approaches offer enhanced safety and efficacy, leading to more precise and aesthetically pleasing outcomes for patients seeking buttock enhancement.

The advancements in gluteal augmentation techniques not only reflect the contributions of key individuals but also the ongoing research and development in the field, which will be discussed further in this work, detailing each method's features, advantages, and disadvantages.

The purpose of this study is to evaluate the various techniques for gluteal augmentation, highlighting their effectiveness, safety, and suitability for different patient profiles. As gluteal augmentation has evolved significantly over the past few decades, understanding the advantages and disadvantages of each method is crucial for informed decision making.

## METHODS

In this article, the author presents a comprehensive review of buttock augmentation techniques, focusing on gluteal implants, Brazilian butt lift (BBL), intramuscular PMMA, and deep subcutaneous hyaluronic acid fillers. Preparation for this review involved an extensive search through peer-reviewed literature, clinical studies, and expert opinions collected from online databases and reputable medical journals. We began by conducting a systematic search for relevant articles that detailed the historical development, advantages, and disadvantages of each augmentation technique. Key terms such as “gluteal implants,” “Brazilian Butt Lift,” “PMMA fillers,” and “hyaluronic acid fillers” were used to ensure a comprehensive capture of existing knowledge in the field. The review is organized to provide a detailed exploration of each technique. We will first examine the historical context and evolution of gluteal augmentation, highlighting significant and procedural advances. Each technique will then be analyzed for its unique characteristics, including efficacy, safety, recovery times, and patient satisfaction.

In addition, the article will discuss the latest developments and trends in the field, synthesizing findings from multiple studies to present a balanced view of the benefits and drawbacks of each method. Through this structured approach, we aim to equip practitioners and patients with the information they need to make informed decisions about buttock augmentation.

The patient provided written informed consent for the publication and the use of her images. All procedures performed in this study are in accordance with the ethical standards of the Brazilian National Research Ethics Commission (CONEP) and the 1964 Declaration of Helsinki (CAAE protocol no. 86722118.8.0000.5291).

## RESULTS

### Advantages and Disadvantages of Subcutaneous, Subfascial, Submuscular, and Intramuscular Gluteal Implants

#### Subcutaneous Gluteal Implants

**Figure 1.**
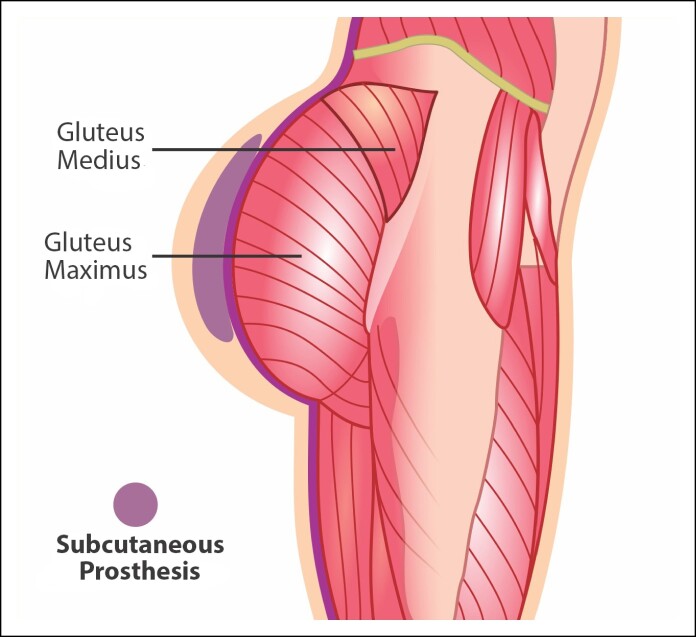
Representation of subcutaneous implant placement in the gluteal region.

Subcutaneous gluteal implants, a breakthrough in cosmetic surgery, offer a way to improve buttock contours. Designed for a natural look, these implants are placed under the skin to provide volume and shape (Figure 1). Ideal for those seeking a more balanced silhouette, their placement is demonstrated in the image above, illustrating the procedure's effectiveness.

Advantages: easier to insert than intramuscular techniques, making it less invasive and quicker; less complexity that may lead to faster recovery and quicker return to activities.

Disadvantages: can be more visible, especially in those with low body fat; risk of a fibrous capsule forming, leading to discomfort and altered shape; fluid accumulation around the implant can occur; higher risk of the implant shifting, causing asymmetry; possible because of trauma or defects, necessitating removal or replacement; increased risk of issues like rippling; slightly higher infection risk because of lower vascularization.

Reasons for decline: the high incidence of complications, such as capsular contractures and seromas, led to the decline in the use of subcutaneous implants; the introduction of implants placed between or within the gluteal muscles has offered a more natural appearance and fewer complications, contributing to the reduced use of subcutaneous implants.

#### Subfascial Gluteal Implants

**Figure 2.**
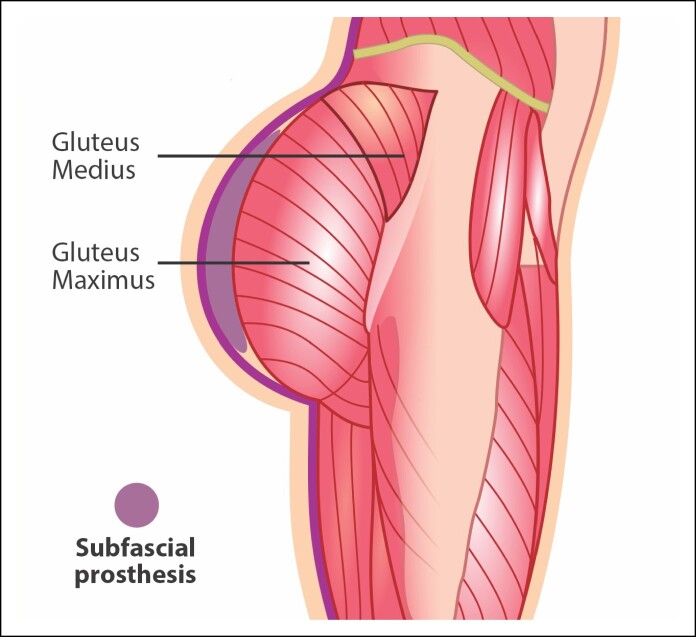
Representation of subfascial implant placement in the gluteal region.

The subfascial implant technique is a method of gluteal augmentation where the implant is inserted beneath the muscle fascia, a connective tissue layer that envelops the muscles (Figure 2). This technique offers an intermediate solution between subcutaneous and intramuscular methods, providing a more natural appearance and reducing some risks associated with other techniques.^16^ Dr Jose Abel de la Peña from Mexico first introduced this technique during a congress held in 1997 and published his findings in 2004.^9,17-20^ The subfascial approach is designed to improve implant stability and the naturalness of the results, and also reduce complications that are often linked with other augmentation techniques.

Advantages: covered by muscle fascia, leading to smoother contours; less likely to shift because of better fixation; minimizes tight fibrous capsule formation; generally results in fewer surface irregularities.

Disadvantages: The subfascial technique is more complex than the subcutaneous approach, requiring greater skill and more surgical time. Recovery may be longer and more painful compared with subcutaneous techniques because of the more invasive nature of the surgery and tissue manipulation. Although less common, there is still a risk of seroma, which is the accumulation of fluid around the implant that may require drainage or other interventions. Implant rupture can occur because of trauma or wear over time. The risk of infection is comparable with that of subcutaneous techniques, necessitating rigorous postoperative care.

These advantages and disadvantages highlight the key considerations when choosing subfascial gluteal implants. The technique offers a more natural appearance and reduced risk of displacement but also presents aesthetic complications and risks associated with a more complex procedure and prolonged recovery.

#### Submuscular Gluteal Implants

**Figure 3.**
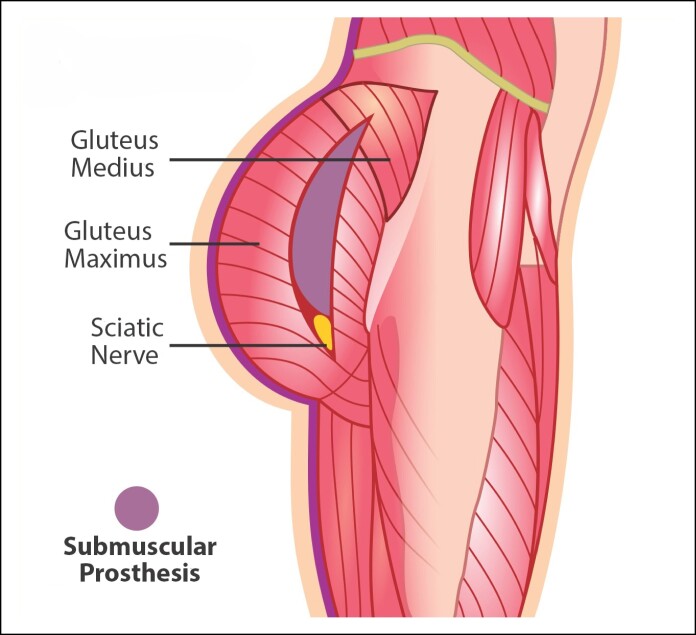
Representation of submuscular implant placement in the gluteal region.

The history of subgluteal implants dates back to the 1980s, marked by the pioneering work of Argentine plastic surgeon Dr José Robles, who first published on the technique in 1984.^8^ Dr Robles is recognized as one of the earliest surgeons to describe and refine the placement of gluteal implants beneath the gluteus maximus muscle (Figure 3). This approach has since become a popular and widely adopted option among plastic surgeons worldwide.^21-23^ It offers patients a safe and effective solution for enhancing the contour and volume of the buttocks, resulting in natural-looking and long-lasting aesthetic outcomes.

Advantages: Submuscular placement provides more natural-looking results. Placing the implant beneath the muscle can lower the risk of capsular contracture and can reduce the risk of implant rupture, especially for patients with active lifestyles. The muscle provides extra protection to the implant, decreasing the likelihood of direct trauma.

Disadvantages: Recovery generally takes longer compared with other implant techniques; because of the manipulation of the muscle during surgery, patients often experience more intense postoperative pain with submuscular placement. There is a risk of nerve injury during submuscular implant placement, which can result in temporary or permanent numbness, tingling, or muscle weakness in the gluteal region.

#### Intramuscular Gluteal Implants

**Figure 4.**
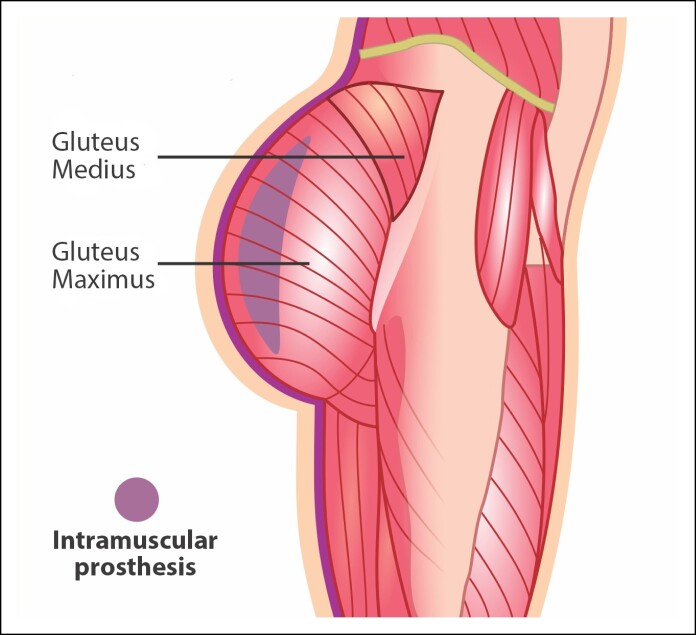
Representation of intramuscular implant placement in the gluteal region.

The intramuscular prosthesis is an advanced technique for gluteal augmentation, where the implant is inserted within the gluteus maximus muscle (Figure 4). This technique is currently one of the most utilized because of its superior aesthetic results, providing a more natural appearance and sensation, as well as lower rates of aesthetic and medical complications. The first author to describe this technique was Dr Rafael Vergara from Mexico, who published his study in 1996 in the journal *Aesthetic Plastic Surgery*.^10^ Subsequently, Dr Raul Gonzalez from Brazil made significant contributions to this technique with the development of the XYZ technique, published in 2004 in the same journal, which optimizes the implant insertion between muscle fibers, providing greater stability and integration of the implant with the muscle tissue.^11^

Advantages: Placing the prosthesis within the muscle provides a more natural appearance and sensation, as the prosthesis is well-covered by muscle tissue, minimizing visibility and palpability. This technique offers greater stability for the prosthesis, reducing the risk of movement and displacement, resulting in a more consistent and symmetrical appearance. The vascularization and constant movement of the muscle help prevent the formation of tight fibrous capsules around the implant, significantly reducing the risk of capsular contracture.

Disadvantages: This technique is more complex and requires greater skill and time from the surgeon. Recovery may be longer and more painful compared with subcutaneous and subfascial techniques because of the greater invasion and manipulation of muscle tissue during surgery. Although lower than with the subcutaneous technique, the risk of seroma is still present. Implant rupture can occur because of trauma or wear over time. Risk of infection is comparable with the subfascial technique. The manipulation of muscle and surrounding tissues may increase the likelihood of infection, necessitating rigorous postoperative care.

The intramuscular gluteal prosthesis technique offers significant advantages in terms of implant appearance and stability, making it a preferred method among many plastic surgeons. Despite the complexity and longer recovery time, the aesthetic benefits and lower incidence of complications make this technique an excellent option for patients seeking natural and durable results in gluteal augmentation.

Table 1 provides a summary of the various types of gluteal implants, outlining their respective advantages and disadvantages. It is important to note that the advantages and disadvantages of each technique may vary depending on the individual characteristics of the patient.

**Table 1.** Different Types of Gluteal Implants, Highlighting Their Respective Advantages and Disadvantages

	Advantages	Disadvantages
Subcutaneous implant	Faster recovery Less postoperative pain Lower risk of muscular complications	Possible visibility of the implant Less implant coverage Higher risk of capsular contracture
Subfascial implant	Additional implant coverage Lower risk of capsular contracture Faster recovery compared with intramuscular	Possible visibility of the implant Less coverage than intramuscular Risk of implant displacement
Submuscular implant	More natural aesthetic results Lower risk of capsular contracture Greater implant coverage Reduced risk of implant rupture	Prolonged recovery time Increased postoperative pain Possibility of nerve impairment
Intramuscular implant	Greater implant coverage Lower risk of implant rupture Reduced risk of migration	Longer recovery time Greater postoperative pain Possible nerve impairment

### Advantages and Disadvantages of Polymethylmethacrylate (Intramuscular), Hyaluronic Acid (Subcutaneous), and Liposculpture for Gluteal Augmentation

Before starting to discuss the different types of fillers, it is important to mention the differences between intramuscular and subcutaneous injections. The discussion about these differences, especially in the context of gluteal augmentation, is crucial, particularly in light of recent research, such as that by Garcia and Pazmino.^24^ These studies highlight the risk of fat embolism (FE) associated with intramuscular injections, which can lead to serious complications. Intramuscular injections are administered directly into the muscle, which is highly vascularized. This increases the risk of fat particles or other materials entering the bloodstream, leading to complications such as FE. On the other hand, subcutaneous injections are given in the fat layer under the skin, where the vascularization is less dense, reducing the risk of serious complications. This reflects an effort to ensure patient safety and discourage practices that could result in serious complications. Therefore, the choice of administration route should be based not only on the desired outcomes but also on patient safety and current legislation.

#### Consideration of Intramuscular PMMA for Gluteal Implants

**Figure 5.**
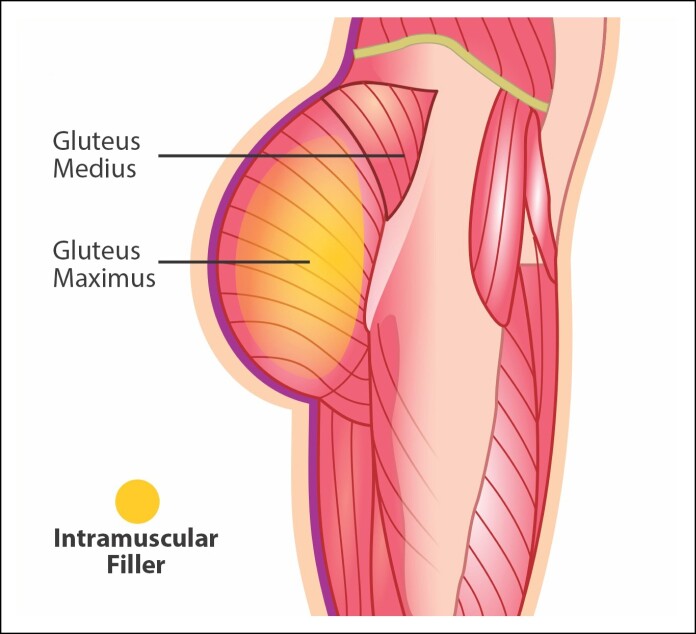
Representation of intramuscular polymethylmethacrylate placement in the gluteal region.

Intramuscular filling for gluteal augmentation is a technique aimed at increasing volume and enhancing the contour of the buttocks through the injection of this substance directly into the gluteus maximus muscle (Figure 5). This approach is less invasive than implant surgery and offers immediate results with a shorter recovery time. In 2019, Dr Chacur published the pioneering article on gluteal augmentation using intramuscular filling. In his study, Dr Chacur and his team reported performing 2770 procedures on 1681 patients over a span of 10 years using PMMA, with a complication rate of 1.88%.^13,14^

Advantages: Less invasive than implant placement, as it does not involve surgery, cuts, or general anesthesia, thus reducing the risks associated with surgery. Recovery is generally faster and less painful, allowing patients to resume their daily activities immediately after the procedure. The amount of filler can be adjusted during the procedure to achieve the desired symmetry and volume, providing greater control over the final results. Patients can see results immediately after the procedure. Risk of severe complications, such as infection and capsular contracture, is significantly reduced.

Disadvantages: Because PMMA is permanent, it is crucial to avoid overcorrection, as removing the product can be extremely difficult. Although rare, there is a risk of adverse reactions or granuloma formation because of the injected material. The amount of volume that can be added is limited compared with implants, which may not be sufficient for patients seeking significant increases. There is an extremely rare risk of material migration during the first week after injection. However, after 2 weeks, the product integrates into the tissue, becoming a permanent substance that no longer migrates. If PMMA is injected superficially into the subcutaneous tissue, visible and palpable nodules may form, highlighting the importance of proper technique to avoid aesthetic complications.

**Figure 6.**
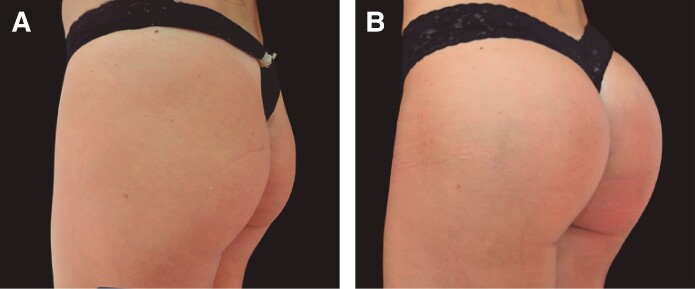
(A) The gluteal region before polymethylmethacrylate injection. (B) The area after (30 min) the procedure, highlighting the natural contour and enhancement. Patient female, 51 years old.

PMMA is often mistakenly equated with illicit substances, such as liquid silicone and hydrogels, which pose significant risks and should not be used for gluteal augmentation. Unlike these dangerous alternatives, PMMA is a well-established material used in medical procedures when administered by qualified professionals. The intramuscular filling technique with PMMA demands meticulous evaluation and the expertise of an experienced practitioner to ensure the procedure's safety and effectiveness.^13-15^ Intramuscular filling for gluteal augmentation with PMMA presents a less invasive alternative to traditional gluteal implants, offering notable benefits, such as quicker recovery times and a reduced risk of severe complications. As a permanent solution, PMMA integrates seamlessly into the muscle tissue, providing lasting support and enhancing the contour of the buttocks, as illustrated in the before-and-after images (Figure 6). This technique is especially suitable for patients seeking moderate improvements in gluteal shape without the need for more invasive surgical procedures. In these procedures reported, we utilized up to 150 mL of PMMA per side, including the anesthetic solution. This volume aligns with a safe approach aimed at minimizing pressure on the tissues and preventing vascular complications, such as embolisms. By employing smaller volumes, along with meticulous technique and the use of fine cannulas (1 mm), we effectively reduce the risk of vascular trauma and associated complications. Literature supports the notion that smaller volumes of filler correlate with a lower incidence of embolic complications, particularly when compared with fat grafting procedures, which typically involve larger volumes and higher tissue pressure.

#### Considerations on Hyaluronic Acid for Gluteal Augmentation

**Figure 7.**
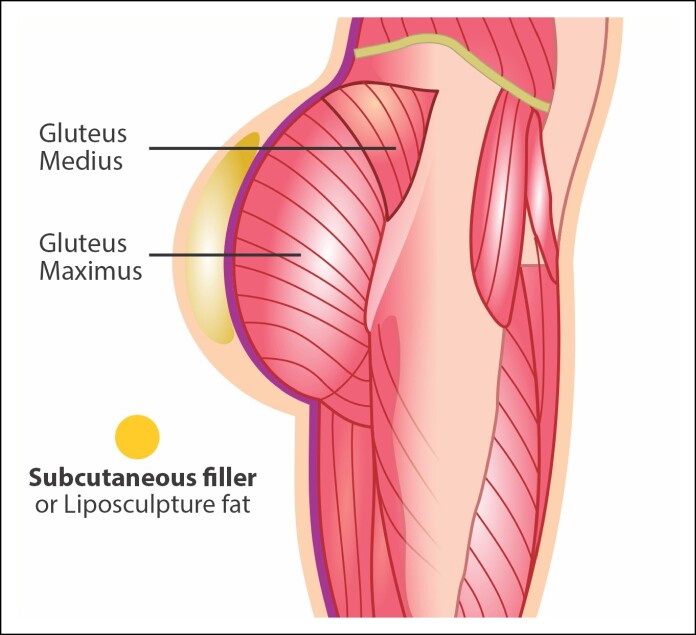
Representation of hyaluronic acid placement in the gluteal region.

Gluteal augmentation with hyaluronic acid is a minimally invasive technique that involves injecting this material to enhance and shape the buttocks. This method is less invasive than surgical implants and offers immediate results with a shorter recovery period. For optimal outcomes, the hyaluronic acid should be administered at a deep subcutaneous level, ideally below the superficial fascia that separates the areolar fat layer from the lamellar fat layer (Figure 7). One of the key studies on gluteal augmentation with hyaluronic acid is published by Santorelli et al.^25^ In that study, the authors evaluated the efficacy and safety of hyaluronic acid for gluteal enhancement in 43 patients, demonstrating favorable aesthetic outcomes and high patient satisfaction. In addition, Robles et al demonstrated that highly crosslinked and redensified hyaluronic acid is a safe and reliable soft tissue filler. It is well tolerated by patients and degrades slowly over time, ensuring long-lasting improvement in the buttocks and cellulite depressions.^26,27^

Advantages: It does not require surgery and reduces risks and recovery time. Patients can see the results immediately after the procedure. Hyaluronic acid can be dissolved if necessary, allowing for adjustments. Reduced risk of infections and complications compared with implants.

Disadvantages: Hyaluronic acid is gradually absorbed by the body, requiring periodic touch-ups; may be more expensive in the long run because of the need for maintenance; and provides less volume increase compared with implants. There is a risk of material migration while the product exists, especially with larger amounts in the same area. Using large quantities in the same application plane can cause subcutaneous detachment, disrupting the cutaneous retinaculum, which are structures connecting the skin to underlying tissues and affecting skin quality.

#### Considerations on Liposculpture for Gluteal Augmentation

Liposculpture for gluteal augmentation, commonly known as the BBL, combines liposuction with fat transfer to enhance the buttocks. This technique provides natural, customizable results while contouring other body areas.^28,29^ Over the years, methods have evolved to improve the survival of transplanted fat. Modern procedures include fat purification through centrifugation, layered injection techniques for even distribution, and creating small tunnels in the recipient tissue to enhance vascularization and integration of the fat. These advancements have increased the survival rate of transplanted fat, resulting in more durable and natural-looking outcomes.^30,31^

The origins of modern liposuction can be traced to Italian gynecologists Arpad and Giorgio Fischer, who developed the first procedure in 1974. For decades, fat grafting was primarily limited to injection and transplantation. Major improvements occurred in 1975, when the Fischers introduced metal cannulas for liposuction.^4^ In 1977, Illouz popularized this technique with improved suction equipment, marking the start of contemporary liposuction tools.^7^ In 1972, German physician Schrudde introduced a less invasive method for removing subcutaneous fat using a uterine curette. By the mid-1970s, other surgeons began using this approach.^32,33^ In 1977, Kesserling and Meyer employed a double-bladed curette with low-powered suction to extract fat separated from deeper tissue, though this method was limited to areas with lower vascularity to reduce complications. Dr Yves-Gérard Illouz, a notable French plastic surgeon, pioneered modern liposuction in 1977 and published his initial studies in 1983, revolutionizing cosmetic surgery and establishing the foundation for contemporary liposculpture.^6,7^

Advantages: using the patient's own fat provides more natural aesthetic results, both in appearance and touch, and improves the contour of other body areas, creating a more harmonious silhouette. There is no risk of rejection or allergic reactions. Removing fat from undesired areas can result in a more sculpted and defined appearance in those regions.

Disadvantages: Part of the transferred fat may be reabsorbed by the body, potentially reducing the initial volume and requiring future touch-ups. It requires the surgeon's skill and experience to achieve balanced results and avoid complications; may be longer, as the patient needs to recover from both the liposuction and the fat transfer. If the fat is not injected evenly, irregularities and asymmetries in the buttocks may occur.

Risk of complications: Although complications such as infections, fat necrosis, and FE can occur, it is important to note that the risks of pulmonary embolism (PE) and FE after BBL procedures are not uncommon. In fact, numerous studies indicate that these complications can lead to death in many cases, not just extreme ones.^24^

Choosing among hyaluronic acid, PMMA implants, liposculpture, and gluteal implants involves evaluating various factors, including patient preferences, aesthetic goals, and the guidance of a skilled plastic surgeon. Each option offers distinct advantages and limitations, which are crucial to consider for achieving the best outcome. Selecting the appropriate method for gluteal augmentation should be a well-informed decision. Factors such as the desired level of enhancement, the permanence of results, the risks involved, and individual health considerations all play a role. An experienced plastic surgeon can provide valuable insights and guidance tailored to each patient's unique needs, ensuring a satisfactory and safe outcome. Table 2 provides a detailed comparison of these techniques, summarizing their main characteristics and differences.

**Table 2.** Comparison of PMMA, Hyaluronic Acid, and Liposculpture Techniques Summarizing Their Main Vantages and Disadvantages for Gluteal Implants

	Advantages	Disadvantages
PMMA	Permanent, substantial volume enhancement	Risks of infection, displacement, or hardening
Hyaluronic acid	Nonsurgical, adjustable, minimal recovery	Short-lived results, potential irregularities
Liposculpture	Natural results, body contouring, no foreign material	Risk of fat embolism, variable fat survival

PMMA, polymethylmethacrylate.

## DISCUSSION

Gluteal augmentation has evolved significantly over the past few decades, offering a range of techniques to cater to individual patient needs and preferences. The primary methods include gluteal implants, liposculpture (commonly known as BBL), intramuscular filling with PMMA, and deep subcutaneous hyaluronic acid fillers. Each technique presents unique features that impact its effectiveness, safety, and suitability for different patient profiles.

Gluteal implants are an effective option for those seeking significant volume increases and predictable results. This technique involves inserting silicone prostheses at various anatomical levels, including subcutaneous, subfascial, submuscular, and intramuscular placements, each with specific advantages and drawbacks. Although they offer immediate and consistent volume enhancement, gluteal implants also carry risks such as infection, displacement, and capsular contracture, which may require future replacements. Additionally, the recovery process tends to be more invasive and prolonged compared with other augmentation methods.

Liposculpture, or the BBL, uses the patient's own fat for gluteal augmentation and contouring. This technique significantly improves fat survival rates, resulting in natural-looking outcomes and enhanced body contouring. It eliminates the risk of rejection associated with foreign materials. However, disadvantages include potential fat reabsorption requiring touch-ups and serious complications like FE, which can lead to PE if fat particles enter the bloodstream.

Intramuscular filling with PMMA is a less invasive technique that involves injecting PMMA directly into the gluteal muscles. This method offers permanent results as PMMA integrates with the muscle tissue, providing continuous support. It is less invasive than implants, reducing associated surgical risks. However, the procedure carries risks of adverse reactions, granuloma formation, and initial material migration. Additionally, removing PMMA can be challenging if necessary.

Deep subcutaneous hyaluronic acid fillers provide a minimally invasive approach for gluteal augmentation. This technique involves injecting hyaluronic acid into the subcutaneous tissue, ideally below the superficial fascia. It offers immediate results and is less invasive than surgical options, with the added benefit of reversibility, as hyaluronic acid can be dissolved if needed. However, limitations include the gradual absorption of the material, which may necessitate periodic touch-ups, and the risk of material migration and potential detachment of the subcutaneous layer with large volumes.

### Limitations

The study has some limitations. The sample size for some procedures may be limited, which affects the generalizability of the findings. In addition, variability in injection techniques and practitioner experience may lead to different results. Many studies may have short follow-up periods, which may not adequately assess long-term outcomes and complications. Heterogeneity between studies in terms of population and outcome measures complicates comparisons. However, despite these limitations, the study underscores the importance of understanding the various techniques and their implications for gluteal augmentation, contributing valuable insights to the field.

## CONCLUSIONS

Each gluteal augmentation technique presents distinct advantages and disadvantages. The choice of method depends on various factors, including patient preferences, available fat for transfer, desired aesthetic outcomes, and the evaluation of an experienced plastic surgeon. Scientific evidence and the surgeon's expertise are crucial in ensuring safe and satisfactory results. It is essential for patients to discuss all available options with a qualified professional to make an informed and personalized decision.
